# Multivariate Analysis of Factors Associated With Axial Symptoms in Unilateral Expansive Open-Door Cervical Laminoplasty With Miniplate Fixation

**DOI:** 10.1097/MD.0000000000002292

**Published:** 2016-01-15

**Authors:** Hua Chen, Hao Liu, Yuxiao Deng, Quan Gong, Tao Li, Yueming Song

**Affiliations:** From the Department of Orthopedics, West China Hospital, Sichuan University, Chengdu Sichuan, P.R. China.

## Abstract

Retrospective case–control study.

Unilateral expansive open-door cervical laminoplasty with miniplate fixation is an efficient and increasing popular surgery for multilevel cervical spondylotic myelopathy. Axial symptoms are the most frequent complaints after cervical laminoplasty. But the mechanisms have not been fully clarified yet.

The objective of this study is to compare the clinical and radiologic data between patients with or without axial symptoms and to investigate the factors associated with axial symptoms by multivariate analysis in cervical laminoplasty with miniplate fixation.

A total of 129 patients who underwent cervical laminoplasty with miniplate fixation were comprised from August 2009 to March 2014. Patients were grouped according to whether they suffered from postoperative axial symptoms (PA) or not (NA). The clinical data including gender, age, duration of symptoms, diagnosis type, medical comorbidity, operative level, blood loss, operative time, pre- and post-Japanese Orthopedic Association (JOA) score, JOA recovery rates, and other complications were recorded. The radiologic data including cervical canal diameter, C2–7 Cobb angle, cervical range of motion (ROM), cross-sectional area, open angle, hinge union, and facet joint destroyed would be measured according to X-ray plain and CT scan images. The univariate analysis and multivariate logistic regression analysis were performed.

There were 39 patients in PA group and 90 patients in NA group. Both groups gained significant JOA improvement postoperatively (*P* < 0.05). The preoperative neck pain (*P* = 0.048), negative change of cervical ROM (*P* = 0.018), and facet joints destroyed (*P* = 0.022) were significant different between the 2 groups. There were no significant differences for other clinical and radiography parameters between the groups (*P* > 0.05). The multivariate analysis showed that the negative change of cervical ROM (OR = 1.062, *P* = 0.047) and facet joints destroyed (OR = 0.661, *P* = 0.024) were related to axial symptoms.

The change of cervical ROM and facet joints destroyed by miniscrews might be associated with axial symptoms after cervical laminoplasty with miniplate fixation. Cervical spine surgeons should carefully operate to decrease the injury of posterior musculature structure and protect the facet joints.

## INTRODUCTION

Unilateral expansive open-door cervical laminoplasty is widely used as a posterior approach surgery to produce efficient decompression and a stable neurologic improvement for multilevel cervical spondylotic myelopathy.^1–3^ Axial symptoms, such as pain and stiffness, were reported to be the most common complaints after cervical laminoplasty.^4,5^ The incidence of axial symptoms has been reported to vary from 6% to 80% in the literatures.^1,4^ Studies have also demonstrated that the postoperative axial symptoms after laminoplasty are significantly higher than those after anterior surgery.^5^ This serious complication greatly impacts patients’ quality of life. The factors leading to axial symptoms are complex. Several possible sources have been proposed, including posterior structures destruction, cervical spinal nerve root damage, facet joint disturbance, posterior muscles dystrophy, cervical lordosis and rang of cervical movement decrease, etc.^4,6–8^ But the mechanisms of axial symptoms have not been fully clarified yet.

In recent years, cervical laminoplasty with miniplate system fixation is becoming popular used in clinical for the miniplate may held the opened laminae securely and prevent the laminae reclosure effectively.^9–12^ Some studies had reported that cervical laminoplasty with miniplate fixation may help preserve more cervical lordosis and range of motion (ROM), avoid facet joints capsule damage by suture suspensory, have a positive effect to induce postoperative axial symptoms.^13,14^ However, there were still 20% to 40% of the patients who underwent cervical laminoplasty with miniplate fixation troubled by this complication and few studies focused on the factors related to axial symptoms, especially for the cervical laminoplasty with miniplate fixation.^13,15^ The objective of the current study was to compare the clinical and radiologic data between patients with or without axial symptoms and to elucidate the potential factors associated with axial symptoms by multivariate analysis in cervical laminoplasty with miniplate fixation.

## MATERIALS AND METHODS

### General Information

This retrospective study was approved by the Ethical Committee of West China Hospital of Sichuan University. All the patients had signed the informed consent form to allow their information to be used for research purposes. From August 2009 to March 2014, a total of 129 patients comprised of 106 male and 23 female with a mean age of 60.6 (range from 31 to 89) years old in the West China Hospital were included. All the patients underwent unilateral expansive open-door cervical laminoplasty with miniplate fixation. The patients who received suture suspensory fixation or skipped miniplate fixation, suffered sudden spinal injury, cervical instability, revision surgery, and hybrid surgery combined with other cervical spine surgery procedures were excluded. All the patients were followed for at least 12 months. Patients who suffered axial symptoms were classified into the postoperative axial symptoms (PA) group, while the others were composed the no-axial (NA) group. There were 39 patients including 30 male and 9 female with a mean age of 62.2 (range from 43 to 79) years old in PA group and 90 patients including 106 male and 23 female with a mean age of 59.8 (range from 31 to 89) years old in NA group.

### Surgical Technique

The patient was positioned prone using a Mayfield three-pin head-holder after general endotracheal anesthesia. The laminae, spinous processes, and medial facet joints from C2 to C7 were exposed. The ligaments between C2 and C3 and between C7 and T1 were cut. Then the spinous processes were amputated from C3 through C7 at their bases. The side with more symptoms was taken as opening side. On the open side, a trough was created using a high speed drill to cut the lamina completely along the junction of the lateral mass and lamina. Then an incomplete fracture hinge was created by removing dorsal cortex and the cancellous bone on the other side. The lamina was carefully opened and an appropriately sized laminoplasty miniplate (Centerpiece Plate Fixation System; Medtronic Sofamor Danek, the United States of America) was chose. The Centerpiece miniplate was anchored to the opened lamina by two 5-mm miniscrews and anchored to the lateral mass by two 7-mm miniscrews. All the patients were encouraged to perform early neck exercises until 2 weeks after the surgeries.

### Clinical Evaluation

The neurological function would be assessed using Japanese Orthopedic Association (JOA) score, and JOA recovery rates were calculated following the formula: recovery rate = (the preoperative JOA score − the final follow-up JOA score)/(17 the preoperative JOA score)∗100%.^16^ The axial symptoms and other complications would be recorded. Axial symptoms were defined as the postoperative pain and stiffness from the nuchal to the scapular region.^17^ The neck and shoulder pain which happened within 1 month after operatives would not be regard as axial symptoms but wound related pain. Additional complications included C5 palsy, cerebrospinal fluid leakage, wound infections, and lung or urinary tract infections were also recorded.

### Radiography Evaluation

**FIGURE 1:**
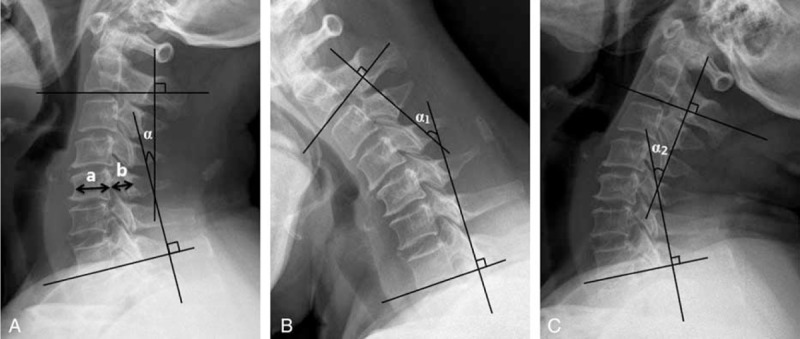
Measurement of anteroposterior diameter (A), C2–7 Cobb angle (A), and cervical range of motion (B, C) b, anteroposterior diameter. Palov's ratio = b/a. α, the C2–7 Cobb angle. Cervical range of motion = α1 + α2.

**FIGURE 2:**
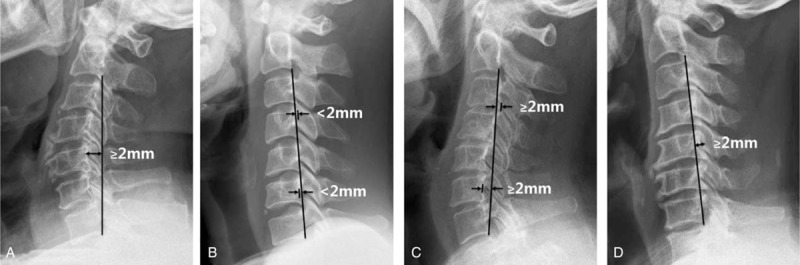
Toyama classification of cervical sagittal alignment: lordosis (A), straight (B), sigmoid (C), and kyphosis (D).

**FIGURE 3:**
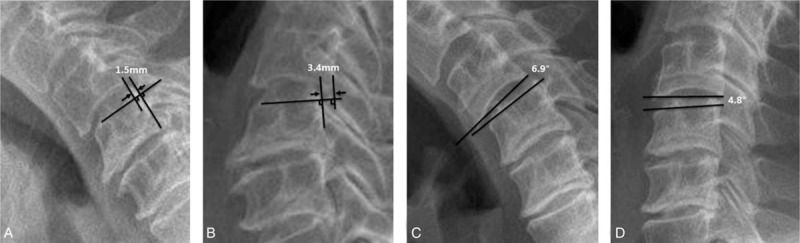
Identification of cervical instability. The slippage displacement of an adjacent vertebra is 4.9 mm (1.5 + 3.4 mm) > 3.5 mm on flexion/extension X-ray radiographs (A, C) or the segmental ROM is 11.7° (6.9° + 4.8°) > 11° (C, D) would be judged as cervical instability.

The anteroposterior (AP) diameter at C5 level, cervical curvature (C2–7 Cobb angle), and cervical ROM were measured using the X-rays plain images (Figure 1), Pavlov's values were also calculated.^18^ Cervical ROM referred to the sum of flexion and extension Cobb angle of C2–C7. Cervical sagittal alignment was morphologically divided to lordosis, straight, sigmoid, and kyphosis according to Toyama classification on neutral lateral X-ray radiographs (Figure 2).^19^ Cervical instability was judged once a slippage displacement of 1 vertebra in relation to an adjacent vertebra on flexion/extension X-ray radiographs is more than 3.5 mm or a segmental ROM is more than 11° (Figure 3).^20^ The cross-sectional area and open angle would be measured using CT images.

**FIGURE 4:**
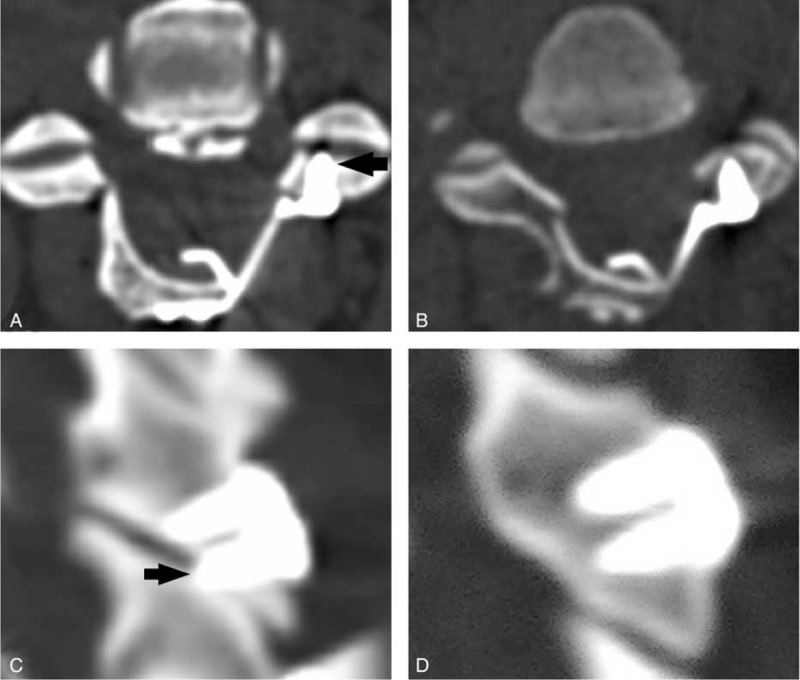
Identification of facet joints damage. (A,C) Facet joints were damaged by the miniscrews (the arrows); (B,D) facet joints kept intact without damaged by the mini-screws.

CT scans data of 1 week postoperatively, including sagittal and axial sectional images, were used to identify the integrity of facet joints. The facet joint could be record as a destroyed facet joint when the articular surface of facet joints was penetrated by the miniscrews which were used to fix the miniplate to lateral mass (Figure 4). The hinge bony fusion was evaluated using 3 and 6 months follow-up CT scans data. Only when the dorsal and ventral cortices of the ends of the hinge fracture both got fused, the hinge bony fusion could be identified.^21^

### Statistical Analysis

Statistical analysis was performed using SPSS version 19.0 software (SPSS Inc., Chicago, IL). Continuous variables with normal distribution were presented as mean ± SD. The paired *t*-tests were performed to detect the difference of preoperative and postoperative data. Univariate analyses including Student's *t*-test and Chi-square test were performed to calculate the differences between PA and NA groups. The variables whose *P*-value was less than 0.10 in univariate analysis would be under considered for multivariate analysis. The multivariate analysis would be performed using multivariate logistic regression analysis. The missing data would not be included in multivariate analysis. A *P*-value of less than 0.05 was considered statistically significant.

## RESULTS

### Clinical Results

The clinical data are summarized in Table 1. The mean follow-up time was 33.3 (range from 12 to 64) months. The JOA score improved significantly from 9.6 ± 2.4 before the surgery to 14.0 ± 2.2 at the final follow-up. The recovery rate was 62% ± 22%. Both the PA and NA groups had significant JOA score improvement postoperatively. Importantly, there were no significant differences in preoperative JOA scores, postoperative JOA scores, and recovery rate between the PA and NA groups (*P* < 0.05). There were no significant differences between the 2 groups in gender, age, diagnosis, duration of disease, medical comorbidity, operative level, blood loss, operative time, and duration of follow-up (*P* < 0.05). However, the different for preoperative neck pain was significant between the 2 groups (*P* = 0.048).

**TABLE 1:**
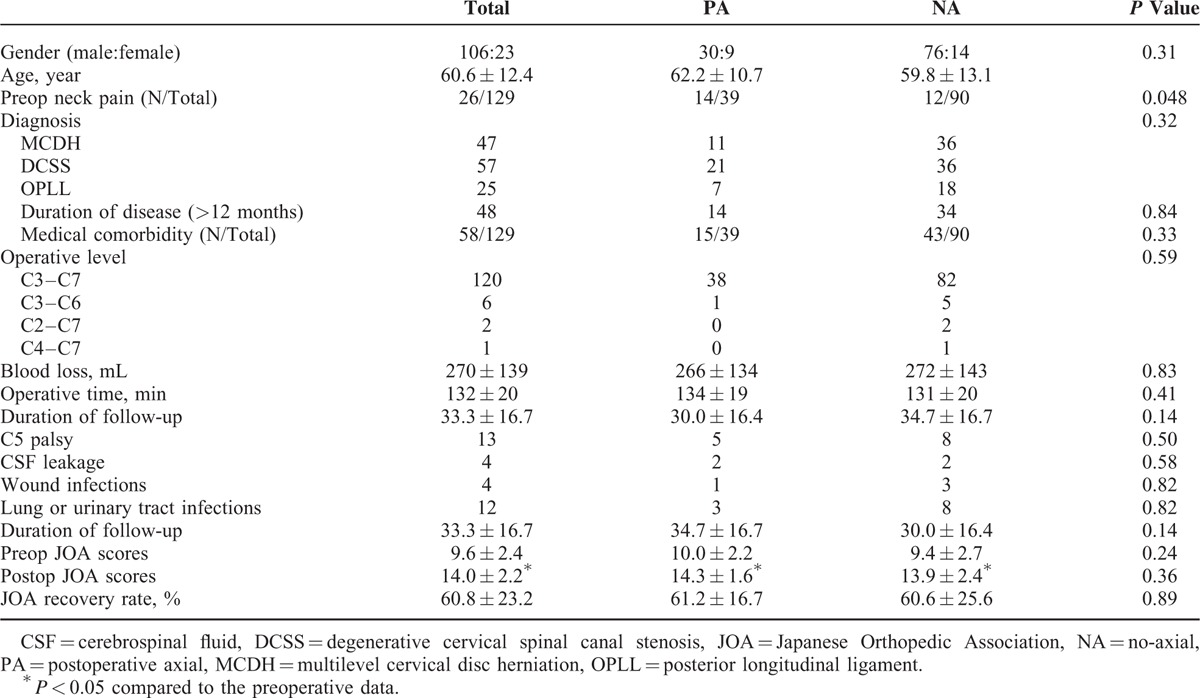
Clinical Factors and Univariate Analysis of Axial Symptom

Thirteen patients experienced C5 palsy, 4 patients had cerebrospinal fluid leakage, 4 patients had wound infections, and 12 patients had lung or urinary tract infections. There were also no significant differences between PA and NA group for these parameters (*P* > 0.05). None plates dislodged or broken, laminar screw back-outs and laminae reclosure were observed during follow-up.

### Radiography Results

**FIGURE 5:**
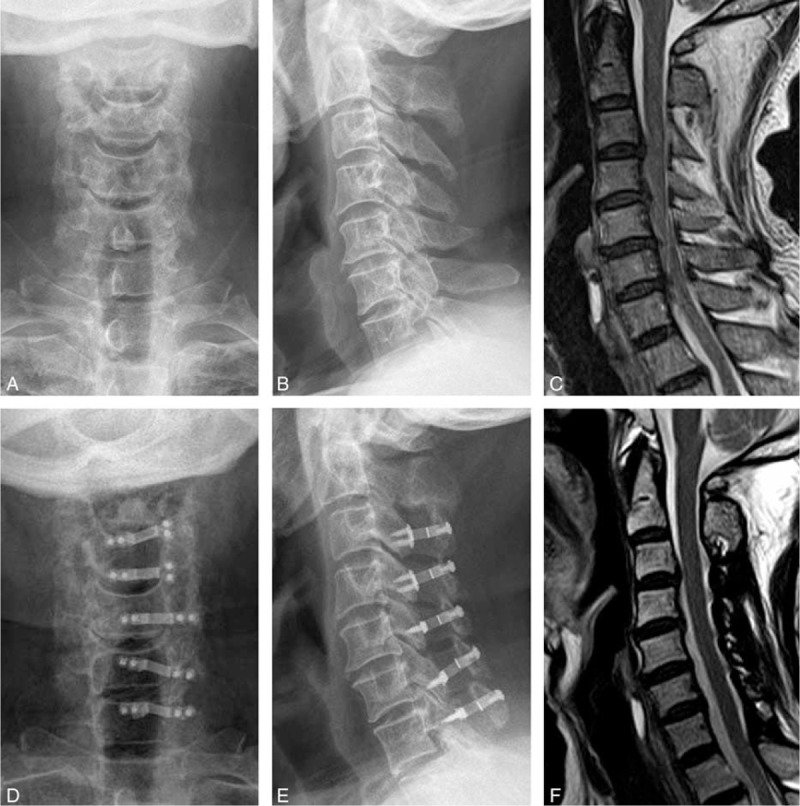
A 65 years old man patients. Preoperative X-ray plain film and middle sagittal film of magnetic resonance showed the cervical spine canal stenosis and the spinal cord compression (A–C). Preoperative X-ray plain film and middle sagittal film of magnetic resonance showed cervical canal expansive, spinal cord decompression and good miniplate fixation (D–F).

The radiography data are summarized in Table 2. C2–7 Cobb angle, AP diameter, Pavlov's value, and Cervical ROM were significantly different before and after surgery for both groups (Figure 5). The differences of these radiologic data between the PA and NA group were not statistically significant (*P* > 0.05). The change of C2–7 Cobb angle (*P* = 0.054), AP diameter (*P* = 0.34), and Pavlov's value (*P* = 0.16) also did not differ significantly between the 2 groups. However, the PA group had greater negative change of cervical ROM than the NA group (*P* = 0.018).

**TABLE 2:**
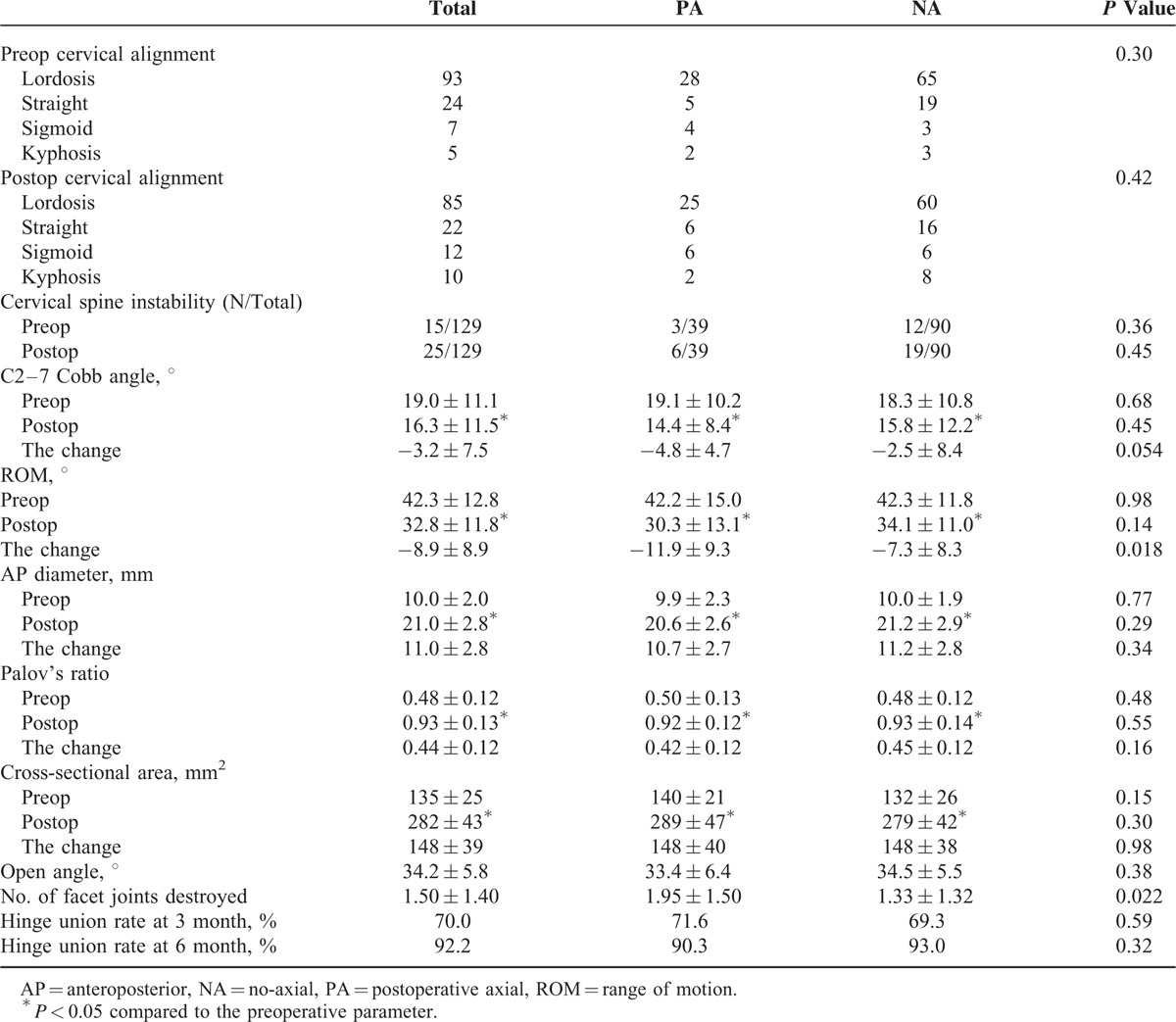
Radiography Factors and Univariate Analysis of Axial Symptom

CT scan data were available for all patients at 1 week postoperative. A total of 110 patients had CT scans data at 3 month follow-up, and 93 patients had CT scans data at 6 month follow-up. There were 196 facet joints destroyed by miniscrews on the open side, as determined by radiologic assessment. The facet joints disturbance rate were 30.6%. The average number of facet joints destroyed in the PA group was statistically significant higher than that in the NA group (*P* = 0.022). The hinge union rate was 70.0% in the PA group and 69.3% in the NA group at 3 months postoperative. It was 90.3% in the PA group and 93.0% in the NA group at 6 months postoperative. The differences of hinge union rate at 3 and 6 months were not significant between the groups (*P* > 0.05).

### Multivariate Analysis

The preoperative neck pain, the change of cervical ROM, the change of C2–7 Cobb angle and facet joint destroyed number were finally included for multivariate logistic regression analysis. The multivariate analysis result showed that the change of cervical ROM (OR = 1.062, *P* = 0.047) and facet joint destroyed number (OR = 0.661, *P* = 0.024) maybe related factors to axial symptoms after cervical laminoplasty with miniplate fixation (Table 3).

**TABLE 3:**
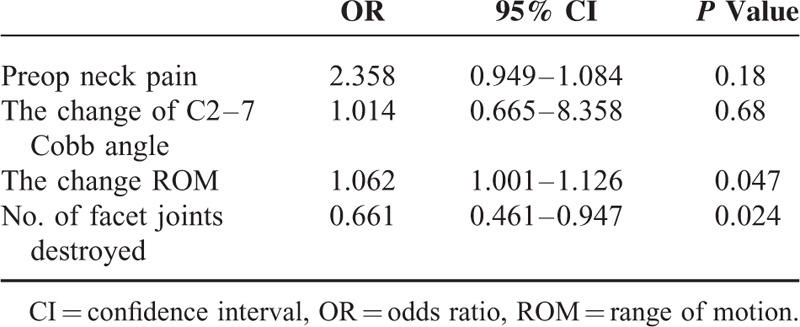
Multivariate Analysis of Factors Associated With Axial Symptom

## DISCUSSION

Unilateral expansive open-door cervical laminoplasty with miniplate fixation was first reported by O’Brien in 1996 and has recently become an increasingly popular method to treat multilevel cervical spondylotic myelopathy.^9–11^ The miniplate fixation system may offer an immediate, rigid fixation for the laminae and stabilize the cervical spinal canal expansion. Axial symptoms are the most common and serious complications after cervical laminoiplasty.^4,5^ Chen et al^13^ reported that cervical laminoplasty with miniplate fixation may reduce postoperative axial symptoms compared to suture suspensory lamioplasty. They proposed that preservation of posterior structure integrity and minimizing facet joints capsule damage ipsilateral to the hinge may contribute to this effect. However, axial symptoms were not completely eliminated in plated cervical laminoplasty. In the Yeh et al^11^ study, moderate to severe neck pain were noted in 42% patients at 3 months after plated cervical laminoplasty. Jiang et al^15^ also reported the axial symptoms rate of plated cervical laminoplasty at 36.7%. These studies, however, did not discuss or elucidate the underlying risk factors for postoperative axial symptoms. In the present study, we found that facet joints damage caused by miniscrews of the miniplate system and negative change of cervical ROM may be associated factors for axial symptoms following cervical laminoplasty with miniplate fixation.

The cervical ROM or the change of cervical ROM was believed to be related to axial symptoms in some literatures.^12,13,22^ However, this problem was still an argued item.^4,23^ In our study, the patients who suffered axial symptoms had a greater reduction in cervical ROM postoperatively. These results suggest that the decrease of cervical ROM may be related to postoperative axial symptoms. The axial symptoms may limit cervical movement. It is difficult to detect whether the loss of cervical ROM is a risk factor for postoperative axial symptoms or a consequence of axial symptoms. Chen et al^13^ has previously reported that axial symptoms were strongly correlated with cervical ROM. Fujimori et al^12^ found that axial symptoms were more strongly associated with loss of the extension angles, but they did not find a correlation between axial symptoms and cervical ROM. The destruction of cervical posterior structures, such as the posterior muscles, nuchal ligament, and other bony structures may lead to the reduction of cervical ROM and, concomitantly, axial symptoms, particularly if the paraspinal muscles are also damaged.^23^ Some researchers^6,7^ suggested that preservation of the muscle insertions of the C2 or C7 spinous process may reduce the persistent postoperative axial symptoms. In our study, we had 6 patients who underwent C3–6 laminoplasty, which preserved the muscle insertions of the C7 spinous process. Indeed, we failed to found a significant relationship between the operative level and axial symptoms. Admittedly, the relatively small sample size of our study may have limited us from detecting a statistically significant effect. The facet joint injury, spontaneous fusion of laminae, and late cervical movement exercise may also be related to the loss of cervical ROM.^23^ Protection of the posterior cervical musculature may help preserve more cervical ROM and reduce axial symptoms, as does early cervical exercise and directed rehabilitation.

Facet joints damage was discussed as another important factor related to axial symptoms. The facet joints may be an isolate pain generator and have been reported implicated in 40% to 55% cases of chronic neck pain.^24^ Wang et al^4^ had reviewed literatures about classic laiminoplasty procedure and concluded that postoperative axial symptoms might be related to dissection around the facets and soft-tissue retraction, necrosis, and scarring. In a neurophysiologic study on facet joints, Cohen et al^25^ reported that capsule stretch activates nociceptors could be a possible cause of persistent neck pain. Therefore, facet capsule suture and stretch may be a possible cause of postlaminoplasty axial symptoms. It is better to keep the facet joint capsule intact to prevent postoperative development of neck pain.^13^

Generally, miniplate could avoid injury of facet joint capsule on the hinge side which was commonly seen in classic suture suspensory fixation cervical laminoplasty. This maybe explains why the miniplate laminoplasty had lower axial symptoms in some studies.^9,13^ Although miniplate can preserve the facet joint intact and prevent joint capsule injury on the hinge side, but a different facet joints damage may occur on the open side unexpectedly. In the present study, we observed the miniscrews used to fix the miniplate to the lateral mass might penetrate the facet joint surface and this situation was quietly common, there were 30.6% miniscrews destroyed facet joints. This facet joint destroy might influence cervical stability and induce nonbacterial inflammation, similar to the facet joint injury on hinge side caused by suture suspensory which may contribute to axial symptoms. This result indicated that implant the miniscrews in cervical lateral mass carelessly might finally increase the axial symptoms after cervical laminoplasty. This problem should not be ignored.

Preoperative neck pain might be related to postoperative axial symptoms. In Yoshida et al^17^ study, the patients who had neck and shoulder symptoms before surgery might continue to have them after surgery, though most of them may get relief of preoperative neck pains after cervical laminoplasty. Ohnari et al^22^ got another result that the preoperative axial symptoms might be not related to postoperative axial symptoms. However, only 31 of 180 patients in his study were included for statistical analysis and the preoperative axial symptoms rate was 59.1% and postoperative axial symptoms rate was 82.3%. The sampling error and a rather high axial symptoms incidence may result in a different conclusion. In the present study, though multivariate analysis showed no significant correlation between preoperative neck pain and postoperative axial symptoms after cervical laminoplasty with miniplate fixation, but univariate analysis got a significant result. This indicated us the patients who had neck or shoulder pain might be a confounding factor to axial symptoms.

Although gender, age, duration of symptoms, cervical alignment, and preoperative cervical ROM were reported to be related to postoperative axial symptoms, our study did not confirm these relationships. Fujibayashi et al^26^ reported that men had a lower axial symptoms rate than women. They suspected that men had stronger neck muscle strength than women might explain the result. In the present study, the incidence of axial symptoms was not significant different between men and women. Kato et al^27^ had reported that older age (>63 years) might have lower risk to suffer axial symptoms. Nevertheless, Yoshida et al^17^ found the incidence of postoperative axial pain was significantly higher in the patient with an age over 70 years old. Ohnari et al^22^ reported that the ages were not significant different between the patients with or without axial symptoms, which was similar with our result.

Hosono et al^5^ reported that hinge nonunion might be a risk factor of axial symptoms after cervical laminoplasty. In the present study, the hinge union rates were not significantly different between the PA and NA groups. Our findings indicate that hinge nonunion in plated cervical laminoplsty may not influence axial symptoms. The rigid fixation of the laminae offered by miniplate system may reduce the micromovements and muscle stimuli caused by nonunion hinge. This may be the reason why the hinge side nonunion did not affect the axial symptoms in our study.

There are some limitations about our study. The present study was a retrospect study. We failed to include more information which may be related to axial symptoms, like the procedure details. The large range of follow-up time and small sample size may lead to the loss of some potential axial symptoms in the further follow-up.

In conclusion, we retrospectively analyzed the clinical and radiologic data between patients with and without axial symptoms after cervical laminoplasty with miniplate fixation. We found that the negative change of cervical ROM and facet joints destroyed by miniscrews might be associated with axial symptoms. Cervical surgeons should carefully operate to decrease the injury of posterior musculature structure and protect the facet joints in surgery.
